# Research on the non-point source pollution of microplastics

**DOI:** 10.3389/fchem.2022.956547

**Published:** 2022-07-22

**Authors:** Li He, Zhongwen Ou, Jiangyang Fan, Boping Zeng, Wei Guan

**Affiliations:** ^1^ Zunyi Normal University College, Zunyi, China; ^2^ Army Logistics University of PLA, Chongqing, China; ^3^ CNOOC Petrochemical Engineering Co., Ltd., Jinan, China; ^4^ Chongqing Key Laboratory of Environmental Materials and Remediation Technologies, Chongqing University of Arts and Sciences, Chongqing, China

**Keywords:** microplastic, non-point source pollution, runoff, drainage basin, water environment

## Abstract

Microplastics are characterized with universality, persistence and toxicity to aquatic organisms, the pollution of microplastics has attracted worldwide attention. At present, studies on microplastic pollution were mainly focused on the composition, abundance and species of microplastics in water bodies and sediments, and few studies were focused on the source and influence characteristics of microplastics in surface water bodies. Starting from the sources of microplastic pollution in surface water of this paper, the pollution status of agricultural microplastics was analyzed, and the importance and urgency of studying microplastic pollution in agricultural non-point sources were put forward. Therefore, it was intended to provide effective scientific basis and technical support for the control of microplastics non-point source pollution in river basins.

## Introduction

Due to the ubiquity, persistence and toxicity of microplastics to aquatic organisms, the pollution problem of microplastics has attracted worldwide attention (Abalansa et al., 2020; Huang et al., 2021). Recently, 125 scientific issues have been raised by scientists on the Frontier of a new age around the world, including finding environmentally friendly alternatives to plastic and managing plastic waste (Anderson et al., 2016; Levine, 2021). The natural degradation of microplastics in the aquatic environment takes centuries, but the microplastics are gradually broken down into smaller substances through physical, chemical and biological reactions. Due to the characteristics of small particle size (particle size is less than 5 mm), high specific surface area, and strong hydrophobicity, microplastics are carriers for long-distance migration of pollutants in different media and have good adsorption effect on pollutants such as antibiotics (Liu et al., 2021; Li et al., 2018a), heavy metals (Zhou et al., 2019; Sarkar et al., 2021) and bacteria (Fueser et al., 2020; Leiser et al., 2021). The combination of microplastics with antibiotics enhances antibiotic resistance genes in the aquatic environment, while, microplastics ingested by biota can negatively affect the physiology, reproduction and immunity of these organisms (Mallik et al., 2021; Mallik et al., 2021; Bertoli et al., 2022), affecting bioaccumulation in the food chain, potentially threatening human health (Prata et al., 2021; Atugoda et al., 2021). The concept of microplastics was proposed by Richard since 2004 (Richard et al., 2004), a large number of related studies have been appeared at home and abroad based on the harmfulness of microplastics. From the perspective of the main body of the polluted environment, the investigation of microplastic pollution was mainly focused on the marine environment, followed by the river, and finally the soil environment. Divided from the categories of pollution sources, the current research was mainly focused on point source pollution such as urban sewage treatment plants, followed by a small number of reports on urban rainwater runoff pollution on major traffic roads. The rainfall process will transfer the microplastics in the soil environment to the surface water through agricultural runoff, combining the microplastic pollution of different environmental subjects. However, there is still a lack of research about agricultural non-point source microplastic pollution.

In summary, this paper started from the sources of microplastic pollution, so the present situation of agricultural microplastic pollution was analyzed, and the importance and urgency of studying microplastic pollution in agricultural non-point sources were put forward. Finally, it provided effectively scientific basis and technical support for the control of microplastics non-point source pollution in river basins.

## Runoff is the main source of microplastics pollution in surface water

The concept of microplastics was proposed by Richard since 2004 (Richard et al., 2004), the microplastic pollution of surface water bodies was mainly concentrated in the marine environment, followed by rivers. At present, researches on microplastic pollution in surface water was mainly focused on the composition, abundance and species of microplastics in water and sediment (Olubukola et al., 2018; McCormick et al., 2014; Grbic et al., 2020). There was few studies have traced the origin and impact characteristics of microplastics in surface water. Sources of microplastics in surface water bodies should include sewage discharge, overflow of combined sewage pipes, rainwater runoff from rural areas and roads, etc. (Dris et al., 2017; Horton et al., 2017; Cheng et al., 2021). There are few existing studies mainly focused on the following two aspects:

1) The abundance of microplastics in the influent and effluent of sewage plants: Mason et al. analyzed the effluent of 17 sewage treatment plants in the United States, and found that the average concentration of microplastics was 0.05 pieces/L, with more than four million pieces/d of microplastics emitted per wastewater treatment plant (Mason et al., 2016). Murphy et al. found that the average concentration of microplastics in the inlet and outlet water of a secondary sewage treatment plant in Scotland was 15.70 and 0.25 pieces/L, respectively. Although the removal rate of microplastics in the sewage treatment plant was as high as 98.41%, the emission of microplastics could still reach 6.5 million pieces/d (Murphy et al., 2016). At the same time, the removed microplastics were discharged back into the environment in the form of residual sludge. Bai et al. (Bai et al., 2018) conducted a study on a sewage plant in Shanghai and found that the microplastic particle size in the inlet and outlet water was mainly 0.36–1.00 mm, and the removal rate of microplastics was 55.6%. The microplastic discharge in the effluent water of the sewage plant was 145.6 billion/d, and the microplastic content in the residual sludge was 540 million/d, which was 40,000 times for the average of the American sewage plant.

All the above studies indicate that the wastewater treatment plant is an important source of microplastics in surface water, and the residual sludge is an important way for the wastewater treatment plant to transfer microplastics pollution. The emission can still reach 6.5 million/d, and the removed microplastics are discharged into the environment again through the excess sludge. Bai et al. (Bai et al., 2018) studied a sewage plant in Shanghai and found that the particle size of microplastics in the influent and effluent is mainly 0.355–1 mm, and the removal rate of microplastics is 55.6%. The microplastic content of the excess sludge is 540 million/d, which is 40,000 times the average of the US sewage treatment plants. All the above studies show that sewage treatment plants are an important source of microplastics in surface water, and excess sludge is an important way for sewage treatment plants to transfer microplastic pollution.

2) Characteristics of microplastic pollution in rainwater runoff from urban roads: Horton et al. (Horton et al., 2017) investigated that British rainwater carried a large amount of plastics such as synthetic fibers, which migrated and accumulated in large amounts to surface water bodies. Chen et al. (Chen H et al., 2020) reported that the total annual load of microplastics from surface runoff, domestic sewage and sewer sediments was almost six times that of wastewater discharge from sewage treatment plants. Bailey et al. (Bailey et al., 2021) indicated that both primary and secondary microplastics could enter the water ecological environment through non-point sources, and urban rainwater runoff carried microplastics related to dust, construction activities, artificial turf, landfill leachate. Tire particles, vehicle debris, or debris from road marking paint produced in the process also contributed to microplastic pollution in urban surface runoff. After decades of research and management, point source pollution has been well controlled. Therefore, non-point source pollution generated by surface runoff is one of the main ways for microplastics to enter water bodies, and the extent of its impact is still unclear.

## Status of agricultural micropastic pollution

Most of the old cities in my country have a combined pipe network, and urban runoff will enter the sewage pipe network, and overflow directly into the river when it exceeds the treatment capacity of the sewage plant, which will have a great impact on the water ecological environment. The use of plastic mulch, agricultural irrigation, and fertilization in the agricultural production process will cause microplastic particles to accumulate in the soil, and enter the river together with the traditional agricultural non-point source pollutants, causing water ecological pollution. Within the scope of the data reviewed, there have been no reports on the overflow of combined sewage pipes and microplastics in agricultural rainwater runoff. Due to the huge contribution of soil to agricultural rainwater runoff pollution, there are many studies on microplastics in soil, mainly focusing on the following aspects:

1) Pollution of plastic mulch: From 2000 to 2016, amount of mulch used in China was increased from 724,000 tons to 1.468 million tons, reaching a peak and accounting for about 70% of the world’s total, covering an area of nearly 1.77 hm^2^ × 10^7^ hm^2^ and accounting for 90% of the world’s total coverage (Bigalke et al., 2021; Bian et al., 2015). With the promulgation of the “Soil Pollution Prevention and Control Plan” and the plastic restriction order, China’s agricultural film production will drop to 774,000 tons in 2020 (Guo et al., 2020). While the use of plastic mulch has declined, plastic concentrations can build up in the soil over time due to its refractory degradation in the environment. When the mulching period in Xinjiang increasing from 5 to 30 years, the content of microplastics in soil was also increased from 91.2 mg/kg to 308.5 mg/kg (Jin et al., 2020).
2) Agricultural irrigation pollution: Since the concentration of microplastics in surface water is 1.00 × 10^−5^ to 1.00×10^5^/L (Olubukola et al., 2018; Mak et al., 2020), the use of surface water and domestic sewage irrigation caused microplastics to re-accumulate in the soil. Bigalke et al. found that microplastic emissions from agricultural drainage in Switzerland were 9.3×10^12^ per year (Bigalke et al., 2021).
3) Fertilization pollution of excess sludge products: According to statistics, the amount of microplastics from sludge fertilization in European farmland was ranged from 63,000 to 430,000 tons each year, and the amount of microplastics in North American farmland was increased from 44,000 to 300,000 tons, which exceeding the pollution concentration of surface seawater (Hao et al., 2021; Lares et al., 2018). The amount of microplastics entering the environment from sludge in China is 1.56×10^14^ per year (65% of which are fibers) (Li et al., 2018b), which is much higher than Iran’s emission of 1.0×10^11^ per year (Petroody et al., 2021). After the accumulation of the above-mentioned ways, the microplastics contained in the soil are 4–23 times more than that in the ocean (Chen H P et al., 2020). According to existing reports, the microplastics in soil are mainly PP, PE, and PVC (Wang et al., 2020). In the Sydney area of Australia, the abundance of soil microplastics was ranged from 300 to 67,500 mg kg^−1^ (Fuller and gautham, 2016). In the Melipira region of Chile, the concentration of microplastics less than 1 mm was ranged from 18,000 to 41,000 kg^−1^ (Corradini et al., 2019). The concentration of microplastics in Yunnan area of China is between 7,000–53,090 kg^−1^ (Zhang and Liu, 2018); The abundance of microplastics in Wuhan plastic film-contaminated vegetable fields is 320–12,560 kg^−1^ (Chen Y et al., 2020); The agricultural plastic film-contaminated vegetables with concentration of microplastics in the ground is 6 × 10^5^ A/kg (Ding et al., 2020). Due to the different industrial and agricultural planting patterns in each country and region, the concentration of microplastics in each region varies greatly. The migration mechanism of microplastics in soil includes vertical migration to deep soil and food chain migration. Among them, the microplastics with smaller particle size have the largest downward movement (Rillig et al., 2017), but it is difficult for the low-density microplastics to migrate downwards, and it is easier to migrate with the runoff (O’connor et al., 2019). However, no microplastics in the soil was flowed to the surface water through the runoff. In order to effectively control microplastic pollution in surface water, it is urgent to study the microplastic pollution process and accumulation mechanism of rural non-point source pollution.

## Status and control methods of microplastics non-point source pollution in river basin

Xie. (2020) studied the application of sewage treatment plant effluent and sludge-based fertilizers in soil on the accumulation of microplastics in the Lijiang River Basin environment. However, due to the lack of data on microplastic pollution from non-point sources in the Lijiang River Basin, the source analysis of the watershed is not complete. Mao et al. (Mao et al., 2020) traced the possible sources of microplastics in the Yulin River, a typical tributary of the Three Gorges Reservoir area, from the perspective of point and non-point source pollution. Urban runoff scouring pollution is the main source of microplastic pollution in Yulin River. However, the authors only sampled and analyzed the soil on the bank slope to speculate the impact of agricultural non-point source pollution, and did not further analyze the microplastic pollution concentration and impact mechanism of rainwater and non-point source pollution.

Zha.(2021) Investigated the distribution characteristics of micro plastics in different non-point source pollution in the Taihu Lake Basin, and found that the average concentration of micro plastics in non-point source pollution from aquaculture, planting and rural domestic sewage was 58.33/(5 L), 14.50/(5 L) and 33.00/(5 L) respectively. The agricultural industry was developed, and the extensive use of agricultural film and chemical fertilizer made the content of micro plastics in local Tiaoxi the highest, reaching 25/(5 L). However, the above studies did not further analyze the relationship between various non-point source pollution and micro plastics in surface water of Taihu Lake.

Zha.(2021) investigated the non-point source pollution control technology on the removal effect of microplastics in Taihu Lake basin, and the results were shown in Table 1. It was found that the process of planting grass ditch, three-stage pre-pond, multi-stage constructed wetland, submerged plant and paddy field could play an obvious role in intercepting microplastics from non-point source pollution. Rural domestic sewage treatment process showed the higher removal effect of microplastics in domestic sewage, which was 79.6%. Construction of wetland and ecological filter bed played an important role in the removal of microplastics, However, there was still a lack of microplastic pollution characteristics and source analysis in the process of non-point source pollution in the watershed, which is consistent with the research of (Daniel and Tony, 2021).

**TABLE 1:** Removal effect of microplastics by different non-point source pollution control technologies.

Treatment technologies	Removal rate of microplastics (%)	Remarks
Control and treatment technology of water and soil, nitrogen and phosphorus in Tiaoxi small watershed	45.8	
Mountain pond restoration and pollutant interception technology	55.6	Planting grass ditch, three-level pre-pond, multi-level artificial wetland
Rice field integrated planting and breeding technology	78.6	
Efficient utilization of nitrogen and phosphorus and system resistance control technology	20	Aquatic plants duckweed and submerged plants
Sewage anaerobic + compound media biological filter for domestic sewage treatment technology	33.3	
Micro-power integrated equipment + ecological filter bed domestic sewage treatment technology	76.5	
Multi-A/O + constructed wetland domestic sewage treatment technology	79.6	
Biological contact oxidation process domestic sewage treatment technology	50	
Domestic sewage treatment technology of A^2^/O + pre-oxidation pond + constructed wetland	62.2	
Three-dimensional integrated oxidation ditch domestic sewage treatment technology	77.1	

## Conclusion

1) Sources of microplastics in surface water included sewage discharge, combined sewer overflows, rural and road stormwater runoff. Sewage treatment plant was an important source of microplastics in surface water, and the residual sludge was a way for sewage treatment plants to transfer microplastics pollution. After decades of research and management, point source pollution had been well controlled. Therefore, non-point source pollution generated by surface runoff was one of the main ways for microplastics entering water, but its degree of influence was not clear.
2) The plastic mulching, agricultural irrigation and fertilization used in agricultural production can cause microplastic particles to accumulate in soil and enter rivers along with traditional agricultural non-point source pollutants, resulting in water ecological pollution. There are no reports about microplastics in the overflow of combined sewage pipes and agricultural rainwater runoff. At present, the accumulation of microplastics in soil is very serious. In order to effectively control the microplastics pollution in surface water, it is urgent to study the microplastics pollution process and accumulation mechanism in rural non-point source pollution process.
3) The pollution characteristics and source resolution process of microplastics in watershed non-point source pollution are not clear and need further study.
